# 

**Published:** 1898-06

**Authors:** 


### MARRIAGES.

At Albany, N. Y., on April 28, 1898, Dr. William Henry Kelly to Miss Sparrow, formerly of Chicago, Ill.

At Philadelphia, June 7, 1898, Dr. Edward M. Ranck and Miss Florence M. Bunch.

### PERSONALS.

Dr. G. Howard Davidson, of Millbrook, will have charge of the cattle at the New York State fair to be held at Syracuse.

Dr. A. W. Bitting, of Lafayette, Ind., has an article in the May 11th *Breeders' Gazette* on "Dipping Sheep for Ticks."

Dr. Leonard Pearson was a visitor to Washington in May, visiting his brother, Mr. R. A. Pearson, of the Dairy Division of the Department of Agriculture.

Dr. W. H. Dalrymple, of Baton Rouge, La., was elected a vice-president, representing the sixth Congressional district at the convention of the Louisiana Sanitary Association in May.

Among the veterinarians noted at the Philadelphia horse-show were Drs. A. W. Clement, Baltimore, Md.; W. H. Ridge, Trevose, Pa.; W. H. Montgomery, Chestnut Hill; S. D. Larzelere, Jenkintown, Pa.; Otto G. Noack, Reading; Leonard Pearson, W. Horace Hoskins, C. J. Marshall, M. W. Drake, S. J. J. Harger, W. N. Fuller, A. F. Schreiber, and Otto von Lang, of Philadelphia.

Dr. F. H. Osgood, of the Massachusetts Light Artillery, has been called to duty, looking to a patrolling of the eastern coast of the Bay State as a safeguard against any Spanish invasion.

Dr. John T. Lee, graduate of the American Veterinary College, located at Tacoma, is President of the State Board of Health and Bureau of Vital Statistics of Washington.

Dr. R. S. Huidekoper, of New York, has been made a commissioned officer in the army, and given the rank of lieutenant-colonel. He will be assigned to duty in charge of one branch of the medical service.

Dr. Leonard Pearson, of Philadelphia, Pa., was an inspector at the horse-show of the Elk Ridge Hunting Club at Baltimore in May.

Veterinarian W. G. Langdon succeeds Dr. T. D. Hinebauch at the North Dakota Agricultural College and Experiment Station.

S. G. Hendron, of York, Pa., graduate of the Veterinary Department of the University of Pennsylvania, has received an appointment under the Bureau of Animal Industry in the meat-inspection service, and directed to report for duty at Indianapolis.

Mr. J. Preston Hoskins, one of the staff of contributors to the JOURNAL, has just been elected to the position of Assistant Professor in German by the Board of Trustees of Princeton University.

Dr. Harry Marshall, Class of '97, Veterinary Department, University of Pennsylvania, is conducting a drug-store in conjunction with his practice at Georgetown, Del.

Dr. W. E. Weihe, formerly of Scranton, Pa., is now associated with the State Board of Agriculture of North Carolina as crop-statistician.

Dr. Wm. Jopling, of Owosso, has served six years as Secretary of the Michigan Association, during which trying period, financially, among the veterinary profession, this association has grown in influence, power, and usefulness, largely attributable to his tireless labors in this direction.

Dr. J. R. Mitchell, of Evansville, Ind., after two days' service in the Bureau of Animal Industry work at Kansas City, resigned his post and returned to his home.

Dr. W. C. Fair, of Cleveland, O., conducts the veterinary department of the *American Sportsman.*

